# Comparison of image quality between Deep learning image reconstruction and Iterative reconstruction technique for CT Brain- a pilot study

**DOI:** 10.12688/f1000research.150773.1

**Published:** 2024-06-26

**Authors:** Obhuli Chandran M, Saikiran Pendem, Priya P S, Cijo Chacko, Priyanka, Rajagopal Kadavigere

**Affiliations:** 1 Department of Medical Imaging Technology, Manipal College of Health Professions, Manipal Academy of Higher Education, Manipal, Karnataka, 576104, India; 2 Department of Radio Diagnosis and Imaging, Kasturba Medical College, Manipal Academy of Higher Education, Manipal, Karnataka, 576104, India; 3 Clinical Scientist, Philips Research and Development, Philips innovation campus, Yelahanka, Karnataka, 560064, India

**Keywords:** Deep learning image reconstruction, iDose4, Image quality, Filtered back projection, CT Brain

## Abstract

**Background:**

Non-contrast Computed Tomography (NCCT) plays a pivotal role in assessing central nervous system disorders and is a crucial diagnostic method. Iterative reconstruction (IR) methods have enhanced image quality (IQ) but may result in a blotchy appearance and decreased resolution for subtle contrasts. The deep-learning image reconstruction (DLIR) algorithm, which integrates a convolutional neural network (CNN) into the reconstruction process, generates high-quality images with minimal noise. Hence, the objective of this study was to assess the IQ of the Precise Image (DLIR) and the IR technique (iDose ^4^) for the NCCT brain.

**Methods:**

This is a prospective study. Thirty patients who underwent NCCT brain were included. The images were reconstructed using DLIR-standard and iDose ^4^. Qualitative IQ analysis parameters, such as overall image quality (OQ), subjective image noise (SIN), and artifacts, were measured. Quantitative IQ analysis parameters such as Computed Tomography (CT) attenuation (HU), image noise (IN), posterior fossa index (PFI), signal-to-noise ratio (SNR), and contrast-to-noise ratio (CNR) in the basal ganglia (BG) and centrum-semiovale (CSO) were measured. Paired t-tests were performed for qualitative and quantitative IQ analyses between the iDose ^4^ and DLIR-standard. Kappa statistics were used to assess inter-observer agreement for qualitative analysis.

**Results:**

Quantitative IQ analysis showed significant differences (p<0.05) in IN, SNR, and CNR between the iDose ^4^ and DLIR-standard at the BG and CSO levels. IN was reduced (41.8-47.6%), SNR (65-82%), and CNR (68-78.8%) were increased with DLIR-standard. PFI was reduced (27.08%) the DLIR-standard. Qualitative IQ analysis showed significant differences (p<0.05) in OQ, SIN, and artifacts between the DLIR standard and iDose ^4^. The DLIR standard showed higher qualitative IQ scores than the iDose ^4^.

**Conclusion:**

DLIR standard yielded superior quantitative and qualitative IQ compared to the IR technique (iDose4). The DLIR-standard significantly reduced the IN and artifacts compared to iDose ^4^ in the NCCT brain.

## Introduction

Computed tomography (CT) is the primary imaging modality used to evaluate patients suspected to have central nervous system disorders. The ability to visualize brain regions quickly and thoroughly is one of their main advantages. This helps in the timely assessment of problems, including stroke, trauma, and intracranial lesions. Non-contrast CT (NCCT) brain scans are widely used in a variety of therapeutic contexts because of their accessibility, speed, and efficacy, which are vital for the early diagnosis and treatment of neurological diseases. ^1^^–^^3^

One notable development in CT reconstruction technology is the use of iterative reconstruction (IR) techniques. iDose ^4^ is a 4 ^th^ generation IR method released by Philips Healthcare that offers improved image quality (IQ) with a reduced radiation dose. IQ and diagnostic precision are improved through IR, which uses sophisticated mathematical techniques to optimize and refine image data. A significant reduction in image noise (IN) allows for clearer visualization of anatomical structures, especially in regions of low contrast, which is the main advantage of the IR technique. IR allows the acquisition of high-quality images with a decreased radiation dose (RD) for patients. For vulnerable groups such as children or those who are radiation-sensitive, this is especially important. ^4^^,^^5^ The IR technique results in a plastic or blotchy appearance at higher reconstruction levels. ^6^^–^^8^

Deep learning image reconstruction (DLIR) algorithms represent a transformative leap in image reconstruction and dose reduction in CT. DLIR in Philips Healthcare is called a Precise Image (PI). Philips PI is the latest and most reliable way to reconstruct high-quality CT images using artificial intelligence (AI) techniques. A trained deep learning neural network was used in the PI reconstruction process. With the fastest reconstruction speed in the market, PI preserves the traditional view of FBP photos. ^9^ By harnessing the capabilities of artificial intelligence (AI), particularly convolutional neural networks (CNNs), these algorithms revolutionize image reconstruction by learning intricate patterns from raw CT data. These algorithms learn complex relationships between sparse or noisy input projections and corresponding standard-dose reference images, enabling the generation of clinically acceptable reconstructions even when using a reduced radiation dose. By leveraging the inherent information within the data and learning intricate patterns, DLIR contributes to the advancement of dose reduction strategies in CT. The DLIR technique yields an image texture reminiscent of FBP, even at low-dose strengths. ^10^^–^^12^ There are limited studies on the usefulness of DLIR on IQ in the NCCT brain. Hence, the aim of this study was to compare the IQ between the new Precise Image (DLIR) and IR (iDose ^4^) techniques for the NCCT Brain.

## Methods

This is a prospective study. The Institutional Ethical Committee (IEC 400/2022) was obtained from Kasturba Medical College and Hospital, Manipal, India, on 1 ^st^ July 1, 2023, followed by the Clinical Trial Registry - India (CTRI) registration (CTRI/2023/07/055310) on 18 ^th^ July 2023. Written informed consent was obtained from all the participants for publication and participation in the study.

**Eligibility criteria**: Thirty patients referred for the NCCT brain were included. Patients referred to the NCCT brain for various clinical indications such as trauma, seizures, stroke, headache, vomiting, fever, chills, and breast carcinoma were included. Patients who were uncooperative and who underwent CT scans with motion artifacts were excluded. The patients included in the study had neuropathological findings on CT, including hemorrhage (n=10), infarct (n=10), tumor (n = 5), VP shunt (n = 1), arachnoid cyst (n = 1), metastases with edema (n=1), cerebral atrophy (n=1), and encephalomalacia (n=1).

**CT Image acquisition:** This study was performed at the Department of Radiodiagnosis, Kasturba Medical College and Hospital. Patients referred for NCCT brain examinations underwent 128 slice CT (Incisive, Philips Healthcare). The technical parameters for the NCCT brain acquisition are listed in Table 1. The images were reconstructed using iDose ^4^ (level 3) ^4^ and the DLIR reconstruction ^9^ level standard.

**Table 1.** Showing the CT technical parameters for Non contrast CT Brain.

Parameter	NCCT Brain
**Tube voltage (kVp)**	120
**Tube current × exposure time (mAs)**	290
**Collimation (mm)**	32 × 0.625
**Rotation time (sec)**	0.5
**Slice thickness (mm)**	3
**Pitch**	0.70
**FOV (mm)**	250
**Matrix size**	512 × 512

**Figure 1.**
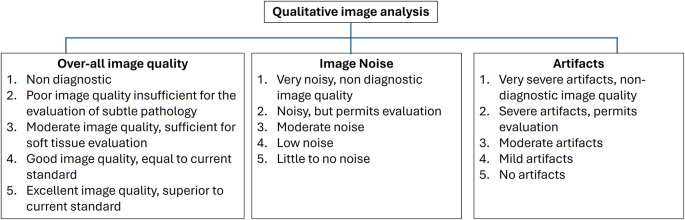
Showing the 5-point Likert scale for qualitative image quality analysis.

**“Qualitative Image quality analysis”:** Two radiologists [reader 1 (R1) and reader 2 (R2)] with over 15 years of experience in neuroradiology imaging evaluated the CT images. Both the readers were blinded to the reconstruction level. The readers assessed the “Overall image quality” (OQ), “Image noise” (IN), “Artifacts” using 5-point Likert scale ( Figure 1).

**Figure 2.**
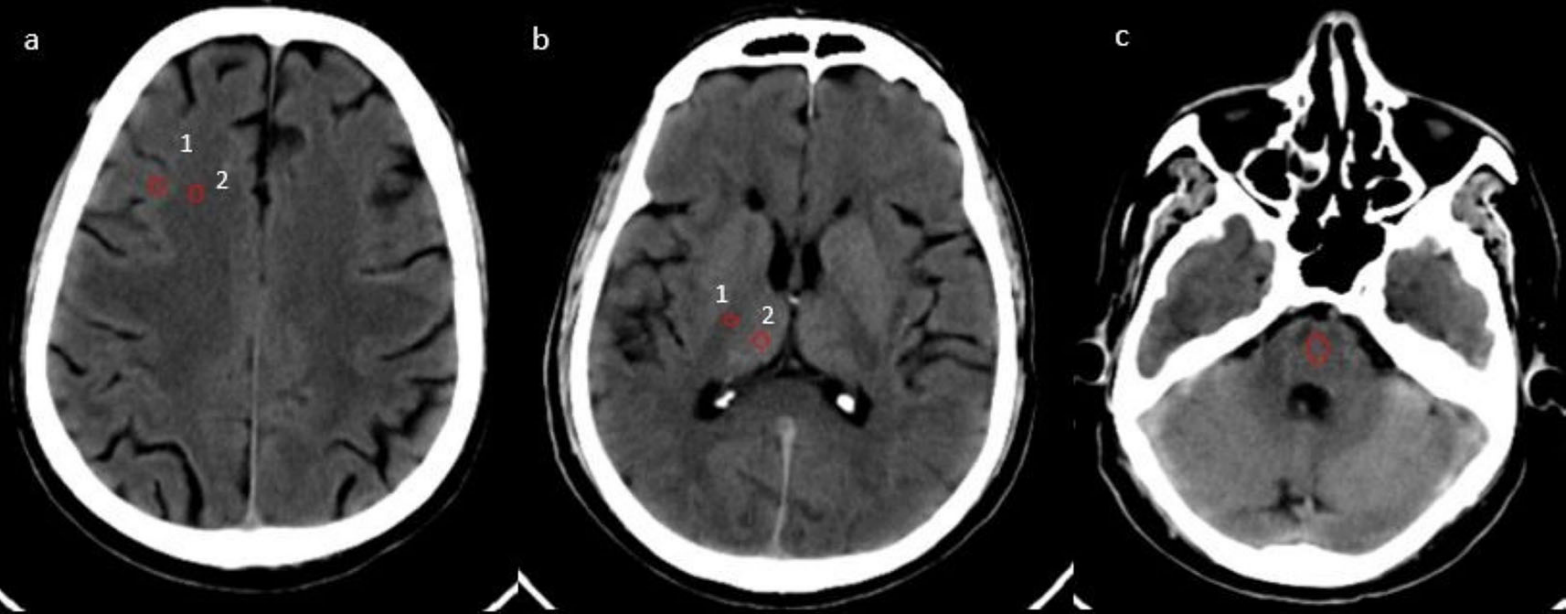
Axial CT images (DLIR-standard) of 61-year old male at the level of centrum semiovale (a) and basal ganglia (b) showing region of interest (ROI) in gray matter and white matter. Axial CT image at the posterior cranial fossa (c) with ROI drawn in the pons region between the petrous bone.

**“Quantitative Image quality analysis”:** CT attenuation (HU) and image noise (IN) of gray matter (GM) and white matter (WM) at the level of the basal ganglia (BG) and centrum-semiovale (CSO) regions were measured. To calculate attenuation (HU) at the GM and WM of the BG, an ROI of 0.1-0.2 cm ^2^ was placed in the thalamus and posterior limb of the internal capsule (PIC). For calculating attenuation at GM and WM of CSO, the ROI of 0.1-0.2 cm ^2^ was placed in the region of frontal WM and adjacent cortical GM ( Figure 2a-b).

The posterior fossa index (PFI) was calculated as the image noise (Standard deviation-SD) of the HU values in the pons. To calculate the PFI, an ROI of 0.2-0.3 cm ^2^ was placed in the pons region of the posterior cranial fossa ( Figure 2c).

The signal-to-noise ratio (SNR) at the BG and CSO levels was calculated as mean CT attenuation (HU)/standard deviation (SD) (SD: Image noise).

The contrast-to-noise ratio (CNR) at the BG and CSO levels was calculated using the following formula:

$$\mathrm{CNR}=\frac{\text{Mean}\;\mathrm{HU}\;\mathrm{GM}-\text{Mean}\;\mathrm{HU}\;\mathrm{WM}}{\sqrt{(\mathrm{SD}}\mathrm{GM})2+\left(\mathrm{SD}\;\mathrm{WM}\right)2}$$

Radiation dose metrics such as “CTDI _volume_ (CTDI _vol_), dose length product (DLP), and size-specific dose estimate (SSDE)” were recorded from the display of the console monitor.

### Statistical analysis

SPSS (IBM, V20.0) was used for statistical analysis. Paired t-tests were performed for qualitative and quantitative IQ analyses between the iDose ^4^ and DLIR-standard. “Kappa (k) statistics” were used to check the interobserver agreement for qualitative analysis. The k-value was considered as follows: <0.20, poor agreement, 0.21-0.40 - Fair agreement, 0.41-0.60 - Moderate agreement, 0.61-0.80 - Good agreement, 0.81-1.00 - Excellent agreement”. Statistical significance was set at P < 0.05.

## Results

A total of thirty patients with 22 males and 8 females with mean age of 55.46±15.38 years referred for NCCT brain were included ( Table 2). The mean CTDI _vol_, DLP, and Size specific dose estimate (SSDE) were 46.36±0.20 mGy, 1157.5±64.23 mGy.cm and 44.10 mGy respectively.

**Table 2.** Showing the characteristics of population.

Characteristics	Mean ± SD
**Age (years)**	55.46±15.38 years
**Gender (%)**	
**Males (n=22)**	73.3%
**Females (n=8)**	26.6%

### Qualitative IQ analysis

**Figure 3.**
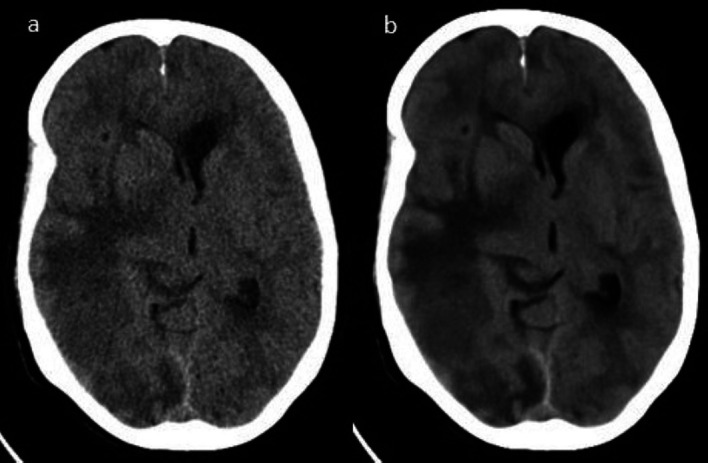
Axial CT images showing the improved image quality with (a) iDose ^4^ compared to (b) DLIR-standard.

Qualitative IQ analysis showed an increase in scores for OQ, IN, and artifacts with DLIR-standard compared to iDose ^4^ for both readers ( Table 3) ( Figure 3a-b).

**Table 3.** Showing qualitative image quality analysis between iDose ^4^ and DLIR-standard.

IQ	iDose ^4^			DLIR-standard			p-value
	R1	R2	k	R1	R2	k	
**OQ**	3.2±0.4	3.2±0.4	0.902	4.2±0.49	4.13±0.43	0.821	<0.05
**IN**	3.03±0.41	3.03±0.41	1.00	4.16±0.46	4.23±0.53	0.837	<0.05
**Artifacts**	3.3±0.59	3.33±0.47	0.80	4.23±0.67	4.26±0.63	0.943	<0.05

OQ - Over all image quality, IN - Image noise, DLIR - Deep learning image reconstruction.

The OQ showed a significant difference (<0.05) between the iDose ^4^ (3.2±0.4; R1) and DLIR-standard (4.2±0.49; R1). IN showed a significant difference (<0.05) between iDose ^4^ (3.03±0.41; R1) and DLIR-standard (4.16±0.46; R1). Artifacts showed significant differences (<0.05) between iDose ^4^ (3.3±0.59 R1) and DLIR-standard (4.23±0.67 R1) ( Table 3).

### Interobserver agreement

For OQ, the agreement between the readers was excellent for iDose ^4^ (0.902) and the DLIR-standard (0.821). For IN, the agreement between readers was excellent for iDose ^4^ (1.00) and the DLIR-Standard (0.837). For artifacts, the agreement between the readers was good for iDose ^4^ (0.80) and excellent for the DLIR-Standard (0.943).

### Quantitative IQ analysis

CT Attenuation (HU) at the BG and CSO levels did not show significant differences (<0.05) in the GM thalamus, WM PIC, adjacent cortical GM, and frontal WM between the iDose ^4^ and DLIR-standard ( Table 4). IN showed significant differences (<0.05) between iDose ^4^ and DLIR-standard at the BG level (GM thalamus, WM PIC) and CSO level (adjacent cortical GM, frontal WM). IN showed 42.8% and 43.47% decreases in GM thalamus and WM PIC, respectively, with DLIR-standard compared to iDose ^4^. IN showed 41.86% and 47.61% decrease in adjacent cortical GM and frontal WM, respectively, with DLIR-standard compared to iDose ^4^. PFI showed significant difference (<0.05) between iDose ^4^ and DLIR-standard with 27.08% IN reduction in the pons region with DLIR-standard compared to iDose ^4^. SNR at BG and CSO levels showed significant differences (<0.05) for the GM thalamus, WM PIC, adjacent cortical GM, and frontal WM between the iDose ^4^ and DLIR-standard. SNR showed 67.60% and 76.78% increases in GM thalamus and WM PIC, respectively, with DLIR-standard compared to iDose ^4^. SNR showed 65% and 82.81% increases at adjacent cortical GM and frontal WM, respectively, with DLIR-standard compared to iDose ^4^. CNR at BG and CSO levels showed significant differences (p < 0.05) in GM thalamus and WM PIC differentiation, adjacent cortical GM, and frontal WM differentiation between iDose ^4^ and DLIR-standard. CNR showed 68% and 78.8% increases in BG and CSO, respectively, with DLIR-standard compared to iDose ^4^.

**Table 4.** Comparison of quantitative image quality analysis between iDose ^4^ and DLIR-standard.

Quantitative parameter	iDose ^4^	DLIR-standard	p-value
**Attenuation (CT HU)**			
**Basal ganglia level**			
GM Thalamus	33.43±1.90	33.41±1.72	>0.05
WM PIC	25.04±2.04	25.07±1.98	>0.05
**Centrum semioval level**			
Adjacent cortical GM	33.26±1.93	33.25±1.65	>0.05
Frontal WM	25.8±2.20	25.7±2.01	>0.05
**Image noise (IN) HU**			
**Basal ganglia level**			
GM Thalamus	4.9±1.06	2.8±0.61	<0.05
WM PIC	4.6±0.93	2.6±0.54	<0.05
**Centrum semiovale level**			
Adjacent cortical GM	4.3±0.94	2.5±0.46	<0.05
Frontal WM	4.2±0.96	2.2±0.40	<0.05
**Posterior fossa index**	5.2±1.41	3.74±1.07	<0.05
**SNR**			
**Basal ganglia level**			
GM Thalamus	7.1±2.12	11.9±2.18	<0.05
WM PIC	5.6±1.22	9.9±2.04	<0.05
**Centrum semiovale level**			
Adjacent cortical GM	8.1±2.22	13.3±2.24	<0.05
Frontal WM	6.4±2.1	11.7±2.5	<0.05
**CNR**			
**Basal ganglia level**			
GM thalamus-WM PLIC	1.25±0.40	2.1±0.58	<0.05
**Centrum semiovale level**			
Adjacent cortical GM-Frontal WM	1.23±0.60	2.2±0.92	<0.05

GM - gray matter; HU - Hounsfield unit; WM - White matter; PIC - Posterior limb of the internal capsule.

## Discussion

In the present study, we compared the qualitative and quantitative IQ between the DLIR-standard (Precise Image) and IR (iDose ^4^) techniques for the NCCT brain. Our study noticed that both qualitative and quantitative IQ improved significantly with the DLIR-standard compared with the iDose ^4^. The new DLIR technique, Precise Image, outperformed the IR technique (iDose ^4^). Our study found that the DLIR standard showed a significant reduction in IN and an increase in SNR and CNR at BG and CSO levels. The DLIR-standard showed higher subjective IQ scores with excellent agreement between readers compared to the iDose ^4^. The lower IN, higher SNR, and CNR might allow for lowering the radiation dose with the DLIR-standard compared to iDose ^4^ for the NCCT brain.

Studies by Kim et al. ^13^ and Alagic et al. ^14^ showed 24-52% and 3.5-43% reduction in IN for NCCT brains with DLIR (True Fidelity; GE) reconstruction levels of low, medium, and high compared with ASIR-V (Adaptive statistical iterative reconstruction-Veo) at BG and CSO levels. DLIR-standard in the present showed a 41.8-47.6% reduction in IN at BG and CSO levels, which is similar to the results of Kim et al. ^13^ and Alagic et al. ^14^ Another Two studies by Oostveen et al. ^15^ and Cozzi et al. ^16^ reported a 9.6% and 13% reduction in IN for NCCT brains with DLIR (AiCE) compared with “hybrid-iterative reconstruction (Hybrid-IR)” and “model-based iterative reconstruction” (MBIR), “Adaptive iterative dose reduction” (AIDR-3D), which is slightly less IN reduction compared to our study. The slight variation in the reduction of IN across CT vendors might suggest the need for further research comparing different reconstruction algorithms.

For the NCCT brain, diagnostic evaluation of the posterior fossa in emergency situations to identify hemorrhagic and ischemic events is important. However, the posterior cranial fossa often experiences beam hardening, streak, and partial volume artifacts due to the presence of bony structures surrounding the cerebellum, pons, and medulla, which leads to diagnostic challenges in identifying hemorrhage and infarct in this region. The artifact index could indicate the extent of CT number fluctuations resulting from artifacts, along with intrinsic image noise linked to factors related to both the scanner and the patient. ^17^^,^^18^ Our study noticed a 27.08% reduction in IN and artifact index in the pons region of the posterior fossa with DLIR-standard compared to iDose ^4^ which was similar to the artifact index reported by Kim et al. ^13^ (17-38%) and Alagic et al. ^14^ (6.8-32.8%). However, Cozzi et al. ^16^ reported a higher artifact index (median 8.4; interquartile range 7.3-9.2) with DLIR (AiCE) than with AIDR-3D (median 7.5; interquartile range 6.9-8.3) in thin sections, which is contrary to the study reported by Oostveen et al. ^15^ with the same DLIR technique. The reason for this could be the difference in the placement of the ROI and slice thickness used between the two studies.

Our study observed higher SNR (65-82%) at BG and CSO levels with DLIR-standard, which suggests better gray and white matter differentiation compared to iDose ^4^. The findings of our study were similar to the results of Alagic et al. ^14^ (2-89%) with DLIR-low, medium, and high levels, and Pula et al. ^19^ (46-59%) with DLIR-High compared to IR techniques. A study by Oostveen et al. ^15^ reported a slightly lower reduction in SNR (5-26%) compared to our study because of the difference in the formula used for calculating the SNR. Our study observed an increase in CNR (68-78.8%) with DLIR-standard at BG and CSO levels, which suggested better gray and white matter differentiation compared to iDose ^4^. The results of our study were similar to the findings reported by Alagic et al. ^14^ (2.4-53%) and Cozzi et al. ^16^ (28-39%). However, Kim et al. ^13^ reported an increase in CNR of 99% with DLIR-high DLIR.

Our study found no significant difference (p>0.05) in CT attenuation (HU) between DLIR-standard and iDose ^4^ at the BG and CSO levels. The findings of our study are similar to the results of Kim et al. ^13^ which showed no significant difference in CT attenuation of GM between DLIR levels and IR technique. However, a study by Alagic et al. ^14^ reported significant differences in CT attenuation between DLIR levels and IR technique at the PLIC WM and adjacent cortical GM, which might suggest that DLIR could lead to minor changes in attenuation values; however, this finding is unlikely to have significant clinical consequences.

The DLIR-standard showed higher qualitative scores for OQ, IN, and artifacts compared to iDose ^4^ which is similar to the findings reported by Oostveen et al. ^15^ and Pula et al. ^19^ Studies by Kim et al. ^13^ and Alagic et al. ^14^ reported an increase in qualitative scores with an increase in the strengths of DLIR from low to high compared to IR techniques.

Our study has a few limitations. First, the study involved a small sample size, and it is necessary to conduct further research with a larger patient cohort to confirm our study findings. Second, the study did not directly evaluate the diagnostic efficacy, which is a crucial step in understanding the complete clinical advantages of DLIR. Third, CT scanning was performed using a standard dose protocol, making it challenging to directly ascertain the potential dose-reduction benefits of DLIR.

## Conclusion

The New DLIR Precise Image (DLIR) technique offers improved image quality with reduced image noise and higher SNR and CNR than iDose ^4^. The DLIR standard also showed higher qualitative image quality scores than the iDose ^4^. The reduction of posterior fossa artifacts with the DLIR standard for the NCCT brain improves the diagnostic accuracy of identifying hemorrhages/infarcts in emergency cases. Our current study may provide implications for performing low-dose scans with reduced radiation doses using DLIR in the NCCT brain.

## Ethics and consent

The Institutional Ethical Committee (IEC 400/2022) was obtained from Kasturba Medical College and Hospital, Manipal, India, on 1 ^st^ July 1, 2023.

Written informed consent was obtained from all the participants for publication and participation in the study.

## Data Availability

Figshare: F1000 Data DLIR NCCT Brain, https://doi.org/10.6084/m9.figshare.25658829.v7. ^20^ This project contains following underlying data:
• Anonymous brain (CT images of all 30 patients -JPEG images) • F1000 Final excel (demographic characteristics of patients, Qualitative and Quantitative analysis - spreadsheet) Data are available under the terms of the Creative Commons Attribution 4.0 International license (CC-BY 4.0).
