# Supplementary material for: Comparison of image quality between Deep learning image reconstruction and Iterative reconstruction technique for CT Brain- a pilot study
Source: F1000Res. 2024 Jun 26;13:691. [Version 1] doi: 10.12688/f1000research.150773.1 (PMC11221345; doi:10.12688/f1000research.150773.1)
Supplement: Peer review history

# Reviewer report: Reviewer response for version 1

**DOI:** 10.5256/f1000research.165371.r296712

**Published:** 2024-07-11

**Authors:** Mustapha Barde

**Affiliations:** 1 Bayero University, Kano, Kano, Nigeria

The article revealed the effectiveness of deep learning iterative image reconstruction (DLIR) as compared to iDose4 (iterative reconstruction) in terms of qualitative and quantitative image quality (IQ) in non-contrast brain CT studies. DLIR presented better signal-to-noise ratio, contrast to noise ratio and reduced image noise. it enhances radiation dose optimization by using low radiation dose for purpose of image acquisition.

 The article is worthy of being published upon addressing the below minor corrections

 Minor corrections

1. The abbreviation FBP used in the introduction and VP in the eligibility criteria part should be written in full for the first time, and subsequently they can be used.
2. In figure 2. The numbers 1 and 2 (ROIs) should be defined on either the image or the figure description.
3. Formula should be written in a standard format, where superscripts is required, they should be correctly inserted.
4. Based on the qualitative IQ analysis results of the different observers, the second observer R2 results should be explained with the corresponding P-value as well, as shown in Table 3. Try to modify the table to indicate the P-values based on each observer.  
5. Quantitative image analysis: the CT attenuation (HU) reported p-value in the result, did not correspond to Table 4. check for Possible sign error in the p-value stated.

Is the work clearly and accurately presented and does it cite the current literature?

Yes

If applicable, is the statistical analysis and its interpretation appropriate?

Yes

Are all the source data underlying the results available to ensure full reproducibility?

Yes

Is the study design appropriate and is the work technically sound?

Yes

Are the conclusions drawn adequately supported by the results?

Yes

Are sufficient details of methods and analysis provided to allow replication by others?

Yes

Reviewer Expertise:

Image analysis and processing, CT Dosimetry

I confirm that I have read this submission and believe that I have an appropriate level of expertise to confirm that it is of an acceptable scientific standard.

# Reviewer report: Reviewer response for version 1

**DOI:** 10.5256/f1000research.165371.r296706

**Published:** 2024-07-03

**Authors:** Shashi Kumar Shetty

**Affiliations:** 1 NITTE university, Karnataka, India

Please find the comments for the article

**Major comments**

**Introduction: **Well-structured. It explained the current reconstruction techniques of CT available in the market and the disadvantages of iDose ^4^. Deep learning-based reconstruction techniques are currently being validated for various applications in CT for image quality improvement and radiation dose reduction. Hence the aim and research gap addressed here is very much relevant to the current context of issues to be addressed while using DLIR techniques for clinical use.

**Methodology: **It clearly explains the key concepts such as inclusion, exclusion criteria, qualitative and quantitative image analysis.

**Statistical analysis: **The tests used for comparing the image quality measures and interobserver agreement appear appropriate.

**Results: **Well-explained with descriptive statistics as well as p-values. The tables provided are informative and easy to understand.

**Discussed: **Clearly explained the results and implications of DLIR in image noise and artifact reduction with clinical relevance.

**Conclusion: **It summarizes the key findings and applications of new precise image technique in clinical aspect for CT brain

**Minor comments**

 Are there any additional costs required for installing these DLIR techniques in CT machines?

 Abbreviations needs to be provided at its first use

****

 Overall, the original research paper provides a significant contribution to the field of advancements in image reconstruction techniques in CT.

Is the work clearly and accurately presented and does it cite the current literature?

Yes

If applicable, is the statistical analysis and its interpretation appropriate?

Yes

Are all the source data underlying the results available to ensure full reproducibility?

Yes

Is the study design appropriate and is the work technically sound?

Yes

Are the conclusions drawn adequately supported by the results?

Yes

Are sufficient details of methods and analysis provided to allow replication by others?

Yes

Reviewer Expertise:

Medical imaging, radiography, radiation protection

I confirm that I have read this submission and believe that I have an appropriate level of expertise to confirm that it is of an acceptable scientific standard.

# Reviewer report: Reviewer response for version 1

**DOI:** 10.5256/f1000research.165371.r296708

**Published:** 2024-07-03

**Authors:** Dr. Tamijeselvan S

**Affiliations:** 1 College of MRIT, Mother Teresa Postgraduate and Research institute of Health sciences, Indira Nagar, Puducherry, India

In this article the author(s) compared the image quality obtained by 2 reconstruction methods namely DLIR standard and IR technique (iDose4). The findings revealed that DLIR-standard gives the better quality image by reducing the image noise and artifacts compared to iDose4 in the NCCT brain.

**Major comments**

 This research article provides a comprehensive comparison of image quality parameters, such as diagnostic value, image noise and artifacts. of DLIR over traditional iterative reconstruction methods in Non contrast CT brain examinations. This will benefit the technologist and the radiologist to understand the various benefits using various available reconstruction method in Computed Tomography particularly in the Non contrast CT brain.

 Since this research was done in a mixed method (both qualitative and quantitative), it reveals a valuable result to upgrade the reconstruction algorithm in future. The biggest challenge in avoiding the base of skull artifacts in CT brain examinations is taken into account for the comparison. Any technical advancement which reduces the patient dose and improve the diagnostic value is always useful in diagnostic radiology. At this view this comparison study is very much appreciable and need of the hour.

**Minor comments**

 Method of sample selection (how the researcher select the sample from the population) can be included in the Methods section.

 Instead of 2 radiologist more number can be used to get more appropriate results.

 Otherwise as a whole, this research article provides valuable data which will lead to quality imaging with less patient dose

Is the work clearly and accurately presented and does it cite the current literature?

Yes

If applicable, is the statistical analysis and its interpretation appropriate?

Yes

Are all the source data underlying the results available to ensure full reproducibility?

Yes

Is the study design appropriate and is the work technically sound?

Yes

Are the conclusions drawn adequately supported by the results?

Yes

Are sufficient details of methods and analysis provided to allow replication by others?

Yes

Reviewer Expertise:

Competency based radiography education, CT Studies, Imaging Technology, Radiography, Radiation Physics

I confirm that I have read this submission and believe that I have an appropriate level of expertise to confirm that it is of an acceptable scientific standard.

## References

[1] : Deep learning versus iterative image reconstruction algorithm for head CT in trauma. *Emerg Radiol* .2022;29(2) : 10.1007/s10140-021-02012-2 339-352 10.1007/s10140-021-02012-2 34984574 PMC8917108

# Reviewer report: Reviewer response for version 1

**DOI:** 10.5256/f1000research.165371.r296703

**Published:** 2024-07-02

**Authors:** Jerald Paul

**Affiliations:** 1 Fatima College of Health Sciences, Al Ain, United Arab Emirates

**Major Comments:**

 This is an interesting study dealing with CT image quality using DLIR and iDose ^4 ^for non-contrast CT brain. The study concludes that DLIR showed higher qualitative scores and reduced image noise (41.8-47.6%) with higher SNR (65-82%) and CNR (68-78.8%).

 The innovation of the study is good. The authors clearly describe the scientific rationale or hypothesis of this study in the manuscript.

 Methods section is described well with various image quality measurements and its formula.

 Results explained clearly. Discussion compared the image quality measures from present study with references from literature. Conclusion explained the benefits of DLIR in Non-contrast CT brain.

**Minor comments:**

 The Methods section can include more details about DLIR.

 Reference for using CNR formula can be provided.

 Abbreviations like SPSS in statistical section can be provided.

 The article is very attractive and provides deep insights about deep learning-based reconstruction techniques in CT.

 Â

Is the work clearly and accurately presented and does it cite the current literature?

Yes

If applicable, is the statistical analysis and its interpretation appropriate?

Yes

Are all the source data underlying the results available to ensure full reproducibility?

Yes

Is the study design appropriate and is the work technically sound?

Yes

Are the conclusions drawn adequately supported by the results?

Yes

Are sufficient details of methods and analysis provided to allow replication by others?

Yes

Reviewer Expertise:

CT , MRI, AI based techniques and digital X rays systems

I confirm that I have read this submission and believe that I have an appropriate level of expertise to confirm that it is of an acceptable scientific standard.

# Reviewer report: Reviewer response for version 1

**DOI:** 10.5256/f1000research.165371.r296709

**Published:** 2024-07-01

**Authors:** Senthil Manikandan Palaniyappan

**Affiliations:** 1 Assistant professor, Department of Radiation Physics, Kidwai Memorial Institute of Oncology, Bangalore, India

In recent years, significant advancements have been made in CT image reconstruction technology, particularly with the introduction of deep learning-based image reconstruction (DLIR) algorithms. One notable development is the "Precise Image" by Philips Healthcare, which aims to reduce image noise and enhance overall image quality for Non-contrast CT brain scans.

**Key Findings and Analysis**

 The study provides a comprehensive comparison of image quality parameters, both qualitative and quantitative, which are crucial for radiographers, technologists, and radiologists seeking to understand the tangible benefits of DLIR over traditional iterative reconstruction methods in CT brain examinations.

 A particularly compelling aspect highlighted in the article is the measurement of the posterior fossa artifact index. Historically, posterior fossa beam hardening artifacts have posed challenges in CT brain examinations, particularly in assessing conditions such as posterior fossa contusions and infarcts. The reduction in artifacts observed in DLIR images compared to previous methods like iDose4 represents a significant advancement that aids radiologists in making more accurate diagnoses.

 Moreover, the article suggests that DLIR has the potential to lower radiation doses while maintaining diagnostic quality, which is critical for the safety and effectiveness of follow-up head CT scans.

**Minor Comments and Future Directions**

 While the study is commendable in its depth and scope, minor adjustments such as limiting the use of quotation marks in the manuscript could enhance clarity and readability.

 Future research avenues could explore the application of DLIR in other body parts and in low-dose CT examinations to further elucidate its benefits and effectiveness across different clinical scenarios.

 This research article contributes valuable insights into the applications and benefits of AI-based technologies in CT for improving image quality and optimizing radiation dose. The adoption of AI techniques such as DLIR, as reported in this study, represents a significant advancement in enhancing patient care through more precise diagnostic imaging.

Is the work clearly and accurately presented and does it cite the current literature?

Yes

If applicable, is the statistical analysis and its interpretation appropriate?

I cannot comment. A qualified statistician is required.

Are all the source data underlying the results available to ensure full reproducibility?

Yes

Is the study design appropriate and is the work technically sound?

Yes

Are the conclusions drawn adequately supported by the results?

Yes

Are sufficient details of methods and analysis provided to allow replication by others?

Yes

Reviewer Expertise:

Radiological Physics

I confirm that I have read this submission and believe that I have an appropriate level of expertise to confirm that it is of an acceptable scientific standard.
